# The role of dopamine D3 receptor and cariprazine in substance use disorders: a systematic review

**DOI:** 10.1192/j.eurpsy.2025.965

**Published:** 2025-08-26

**Authors:** B. A. Lázár, A. Anand, J. Hevesi, J. Gajdics, O. Bagi, F. F. Farkas, B. K. Kádár

**Affiliations:** 1 Department of Psychiatry, University of Szeged, Szeged, Hungary

## Abstract

**Introduction:**

In recent years, dopamine D3 receptor (DRD3) has gained extensive attention in substance use disorders (SUDs) in terms of their anatomical localization and role in drug-related processes. Animal studies have shown that DRD3 agonists modulate addictive behaviour. In addition, cariprazine (CAR), a novel antipsychotic with a partial agonist effect on DRD3, may be a treatment option for patients diagnosed with schizophrenia (SCZ) with comorbid SUD.

**Objectives:**

Therefore, the main goal of the present work was to summarize literature data about DRD3 and CAR in SUDs.

**Methods:**

A systematic review was conducted in August 2024. The full-text search was performed without filtering from four databases (PubMed, ScienceDirect, Web of Science, Cochrane Registry). In the first search “dopamine receptor D3” AND “substance use” OR “addiction” OR “dependence” OR “misuse” were used as the key search terms, and in the second search “cariprazine” AND “substance use” OR “addiction” OR “dependence” OR “misuse” were used. Duplicated studies, non-relevant articles, review articles, and animal and cell studies were excluded.

**Results:**

In the first search, 40 articles were identified; however, 15 were excluded. In the second search, 21 articles were identified; however, 12 were excluded. Findings based on the 25 included articles show that DRD3 modulators, which are mostly agonists of the receptors, may have a positive effect on both psychotic symptoms and substance use frequency- and drug-seeking behavioral reduction. Our findings based 9 included articles demonstrate that CAR is a more effective and safe medication for SCZ with comorbid SUD than other atypical antipsychotics. It could also be suggested that in other psychiatric conditions where substance abuse is occurring CAR is also a good treatment option.

**Conclusions:**

Based on past and current research, it’s crucial to systematically evaluate the role of DRD3 for developing new therapeutic perspectives in SUDs, though more research is needed to confirm the efficacy and safety of DRD3 modulators and CAR as medications for SUDs. Furthermore, the present review suggests that CAR may be the optimal antipsychotic for treating SCZ with comorbid SUDs.

**Disclosure of Interest:**

None Declared

